# Parental acceptance and children’s psychological adjustment: The moderating effects of interpersonal power and prestige across age

**DOI:** 10.1371/journal.pone.0215325

**Published:** 2019-04-11

**Authors:** Miguel Angel Carrasco, Begoña Delgado, Francisco Pablo Holgado-Tello

**Affiliations:** 1 Department of Personality, Assessment and Psychological Treatment, Faculty of Psychology, Universidad Nacional de Educación a Distancia (UNED), Madrid, Spain; 2 Department of Developmental Psychology, Faculty of Psychology, Universidad Nacional de Educación a Distancia (UNED), Madrid, Spain; 3 Department of Methodology of the Behavioral Sciences, Faculty of Psychology, Universidad Nacional de Educación a Distancia (UNED), Madrid, Spain; Institute of Physiology and Basic Medicine, RUSSIAN FEDERATION

## Abstract

The differential contribution of maternal and paternal acceptance-rejection to children’s psychological adjustment has been explained by differences in interpersonal power and prestige within families; however, there is not yet enough empirical support for this explanation. This study examines the moderating effects of interpersonal power and prestige on the relationship between perceived parental acceptance-rejection and psychological adjustment across children’s sex and age. The sample was composed of 913 children ranging in age from 9 to 16 years. Multiple hierarchical regression analyses in the total sample showed a significant and independent contribution of parental acceptance-rejection and parental power and prestige. No moderating effects of interpersonal power and prestige were found for the total sample. However, when the regression analyses were conducted across different age groups, maternal acceptance had a higher contribution to psychological adjustment in children from nine to ten years old. Interestingly, the moderating effects of interpersonal prestige (not interpersonal power) were also significant in younger participants. Furthermore, the moderating effects of prestige on maternal acceptance-rejection were different in late childhood than in early adolescence. These results suggest how parental prestige may explain the higher contribution of maternal acceptance to younger children’s psychological adjustment.

## Introduction

Traditionally it has been assumed that children’s psychological adjustment is related to parent-child relationships [1, 2] and, more generally, to the way parents care for their children. From a cross-cultural perspective, the interpersonal acceptance-rejection theory (IPARTheory, [3–4]), formally known as PARTheory, has been supported by much cross-cultural evidence that interpersonal acceptance-rejection is related to individuals’ psychological adjustment. Parental acceptance (by mothers and fathers) is particularly closely associated with children’s psychological adjustment [3, 4, 5–8]. As a kind of natural law, in all analyzed cultures, children’s psychological adjustment has been significantly and positively related to perceived parental acceptance. However, fathers and mothers did not always make the same contribution to children’s psychological problems. In some studies, paternal rejection makes a greater contribution to children’s maladjustment [9, 10], while in others maternal rejection appears to be the most painful for children [11–14]. In the context of the PARTheory, a previous meta-analysis conducted by Khaleque and Rohner [8] showed that the mean weighted effect size of the correlation between perceived paternal acceptance and children’s psychological adjustment was significantly larger than the mean weighted effect size of the correlation between perceived maternal acceptance and children’s psychological adjustment. Thus, although the acceptance-rejection of both parents has important effects on the child’s adjustment, occasionally the contribution of one becomes more relevant than the contribution of another. The present study explores this differential contribution of perceived parental acceptance (fathers versus mothers) on children’s adjustment, taking into account the role of perceived interpersonal power and prestige of mothers and fathers in the familial dynamic. These results, suggest the need to explore possible mechanisms that might explain why the love-related behaviors of one parent sometimes have a significantly greater impact on offspring’s adjustment than the love-related behaviors of the other parent.

Interpersonal power and prestige refer to individual characteristics of parents as perceived by children. These factors come mainly from studies of groups and leadership in the field of social psychology [15–17]. Specifically, interpersonal power is defined as the individual’s capacity to influence the decisions and behaviors of others [16–18]. This ability originates in interpersonal exchanges and is not based on any status, category or level of authority. On the other hand, interpersonal prestige is understood as recognition; it refers to the signs of social approval, esteem, respect and admiration that an individual accord to another person or group of people.

Previous research from different perspectives [19–22] has shown the relevance that parental power and prestige have to children’s psychological adjustment and satisfaction with their family functioning. In the context of IPARTheory, Carrasco and Rohner [23], with a sample of 313 Spanish children aged 9 through 13, found that maternal acceptance, compared to paternal acceptance, affected the children’s psychological adjustment when mothers were perceived to have both higher power and higher prestige than fathers. However, the strongest overall contribution to children’s adjustment was made in families where fathers were perceived to have both the highest power and the highest prestige. In a more recent special issue [24] on 13 studies in 11 nations (Bangladesh, China, Croatia, Greece, Korea, Pakistan, Poland, Portugal, Spain, Turkey and the United Kingdom), the results of these analyses showed that either maternal or paternal power or prestige—or both power and prestige—moderated the relationship between perceived parental (maternal and/or paternal) acceptance and offspring’s adjustment in eight of the studies (62%). In the Spanish sample [25], in particular, both perceived interpersonal power and prestige significantly moderated the relationship between perceived paternal acceptance and children’s psychological adjustment. The relationship between perceived paternal acceptance and children’s adjustment intensified to the degree that children perceived their parents to share power and prestige equally. In addition, the effects of perceived paternal acceptance on children’s adjustment were especially strong when fathers were perceived to have both more interpersonal power and more prestige than mothers. The conclusions of this previous research support the theory that offspring’s perceptions of parental power and prestige constitute one class of variables that helps to explain why the love-related behavior of fathers sometimes fails (in many international contexts) to make a significant contribution to offspring’s adjustment when these behaviors by mothers are controlled. However, we do not know yet why only perceived parental *power* moderates this relationship in some instances, but only perceived parental *prestige* moderates it in others [24].

Furthermore, children’s sex and age are two important variables to be consider for several reasons: (1) many studies of gender differences in perceived parental acceptance are mixed and inconsistent (see [26]); (2) children perceive a decrease in parental warmth, involvement and support as they grow up [27–29]; (3) there is empirical evidence for a normative decrease in behavioral problems as children grow older [30, 31]; (4) few studies have explored sex and age differences in parental power and prestige, and results have been inconsistent [24]. For these reasons it is advisable to consider the sex and age of the child when exploring the two-way interactions of maternal and paternal acceptance by interpersonal power and prestige on children’s outcomes.

The aim of this paper is to analyze the extent to which the interpersonal power and prestige of parents moderate the direct relationship between parental acceptance and children’s psychological adjustment. This objective will be approached from a developmental framework, considering the children’s age and sex. To our knowledge, no studies have compared these relations across different age groups. Any effect between these variables could be sensitive to the cognitive and social advances that occur from late childhood to adolescence, as well to the corresponding adjustments in family functioning.

## Materials and method

The manuscript has been carried out under the norms recommended in research on human subjects by the deontological code of European Community and the American Psychological Association´s Ethical Standards for Research and Publication. The research was approved by the Bioethics Committee of the UNED. Also, we have obtained the corresponding permissions and written consent. We also have guaranteed the privacy in the treatment of data. The participation in the study was voluntary, anonymous, and contingent upon the written consent of his or her parents.

### Participants

The sample consists of 983 Spanish children and adolescents (449 boys) ranging in age from 9 to 16 years (*M* = 13.09; *SD* = 2.00). Participants were selected through simple random sampling from 20 public and publicly funded private schools in 18 different cities in Spain. The participation in the study was voluntary, anonymous, and contingent upon the written consent of his or her parents. For each school, one class was picked at random from each educational level. The majority of the children lived with their biological parents (84.5%), who were employed (85.1% fathers; and 64.6% mothers). Respect the numbers of siblings, the 58% participants lived in families with two.

The inclusion criteria of the sample were, first, have parental consent, and second, submit a fully completed assessment protocol. Most belonged to the ethnic group “white European.” The percentage of students did not get an approval from their parents to participate in the research was 8.5%

### Instruments

- *Parental Power and Prestige Questionnaire*: *Child Version* (3PQ; [32]; adapted to Spanish population by [33]). It consists of ten items designed to assess the perceptions of children about the relative power and prestige of their fathers *versus* their mothers. Five items assess the perception of interpersonal power (e.g., “Whose opinions usually influence you the most?”), and the other five the perception of interpersonal prestige (e.g., “Whom do you personally admire more?”). The items are accompanied by a scale with the following five points: (1) My mother most often; (2) My mother more than my father; (3) My mother and father alike; (4) My father more than my mother; (5) My father most often. The factor scores range between 5 and 25. Scores below 15 (midpoint) reveal the perception that mothers have more power or prestige that fathers. Scores above 15 indicate that fathers are perceived to have more power or prestige than mothers. Scores around the midpoint indicate that father and mother are perceived as having equal power or prestige. For the total scale, scores range from 10 to 50. Scores below 30 (midpoint) indicate that mothers are perceived to have more power and prestige; scores above 30 suggest that fathers are perceived to have more power and prestige than mothers. Scores close to the midpoint reveal that both parents are perceived as having similar levels of power and prestige. The Cronbach’s alpha was 0.78 [33]. In this instrument, the higher the score, the more influence the father has, as opposed to the mother.
- *The Acceptance-Rejection Questionnaire/Parental Control for Children* (Parental Acceptance-Rejection/Control Questionnaire, PARQ/C; [34]; adapted to Spanish population by [35]), short version for mothers (PARQ-M) and fathers (PARQ-F). The short form consists of 29 items of which 5 refer to parental control. Versions completed the child on the mother and the father are identical except that the items relate to the corresponding parental figure. Thus, mother and father versions of PARQ were the same. All items are evaluated by a 4-point Likert scale: 1 “almost never true,” 2 “sometimes true,” 3 “often true,” and 4 “almost always true.” The questionnaire is divided into four subscales: warmth/affection (e.g., “My mother [father] says good things about me”), hostility/aggression (e.g., “My mother [father] hits me even though I do not deserve it”), indifference/neglect (e.g., “My mother [father] does not pay attention to me”), and undifferentiated rejection (e.g., “My mother [father] sees me as a great nuisance”). The sum of these four scales (with the scale of warmth/inverted coldness) provides an added measure of perceived acceptance-rejection, for which scores range from 24 (maximum perceived acceptance) to 96 (maximum perceived rejection). The control scale is assessed independently. Total scores above 60 reveals qualitatively more rejection than acceptance. This instrument has been used in more than 500 studies in different countries. The psychometric properties have been shown to be excellent [7, 32, 34]. The Cronbach’s alpha for PARQF and PARQM was 0.88 [35].
- The *Personality Assessment Questionnaire*: *Child Version* (child PAQ; [36]; adapted to Spanish population by [37]). It consists of 42 items that assess 7 personality provisions: (1) hostility/aggression, passive aggression or problems in managing hostility and aggression (e.g., “I think of hitting or being rude”); (2) dependency or defensive independence on the form, frequency, severity and timing of perceived rejection (e.g., “I want my parents to love me very much”); (3) self-esteem negative (e.g., “When I meet someone I think is better than me”); (4) self-efficacy negative (e.g., “I think I cannot do things right”); (5) lack of emotional response (e.g., “I cannot show others how I feel”); 6) emotional instability (e.g., “I get upset when things go wrong”); and 7) negative view of the world (e.g., “I believe that life is full of dangers”). The items are answered on a 4-point Likert scale ranging from 1 “almost never true” to 4 “almost always true.” The sum of the seven scales provides an aggregate score indicating the degree of psychological adjustment of the child. Scores at or above 105 reveal that the children experience themselves to be more maladjusted than adjusted. This instrument has been widely used and has good evidence of validity and reliability [7, 36]. The Cronbach’s alpha was 0.82 [37].

### Procedure

As part of a larger study on psychological adjustment in children, 18 schools were randomly selected from different cities in Spain. We received authorization to conduct the study from the schools’ administrators. Each child’s participation in the study was voluntary and contingent on the informed consent of his or her parents. The children were asked to complete the measures in the classroom. At the end of the study, parents received feedback regarding the main results.

The instructions to get protocols are available in Protocols.io (http://dx.doi.org/10.17504/protocols.io.zc5f2y6). Please, notice previous authorization is required by the authors to use the original instruments under copyright.

### Statistical analyses

First, a preliminary analysis was carried out in which the correlations between the variables and the basic descriptive of the variables were obtained. Second, five different regression analyses with a hierarchical order of inclusion were conducted. As predictors, the scores obtained in the PARQF, PARQM, and Interpersonal Power and Prestige questionnaire were used, and as dependent variable, the child’s adjustment measured by the PAQ was used. In the first hierarchical regression analysis, sex and age were included as covariables in the first step; the second step included perceived paternal (PARQF) and maternal (PARQM) acceptance-rejection, parental power and parental prestige to test for main effects; and the third step included the product variables to test for possible two-way interactions of maternal and paternal acceptance with interpersonal power and prestige. Four additional regression analyses were conducted, one for each age group: 9–10, 11–12, 13–14 and 15–16 years. In these regression analyses, sex and age were not included. Finally, a post hoc analysis of interactions was conducted using the Johnson‐Neyman technique with the Hayes’s PROCESS command in SPSS [38]. In order to plot the significant interactions, the sample was divided into three different groups considering values for moderator mean and plus/minus one standard deviation from mean. All analyses were conducted using the IBM SPSS statistics 21 software.

## Results

### Preliminary analysis

As a first approach to the analysis of the data, a correlational analysis (Table 1) and a hierarchical regression analysis adjusted by sex and age (Table 2) were conducted for the total sample. Given the large sample size, most correlations are significant; however, highest correlations are found between maternal acceptance-rejection (PARQM) or paternal acceptance-rejection (PARQF) and children’s psychological maladjustment (PAQ), and between parental prestige and parental power. These results show significant positive correlations between children’s psychological maladjustment and perceived paternal and maternal rejection. Thus, the higher the level of perceived parental rejection, the higher the level of children’s maladjustment. In addition, the higher the parental power or prestige, the lower the perceived paternal rejection and the lower children’s maladjustment. According to the mean scores (Table 1), offspring tended to perceive both their mothers and fathers as being loving (accepting). Moreover, children tended to self-report, on average, at least fair psychological adjustment and they also tended to perceive their mothers as having somewhat more power or prestige than their fathers.

**Table 1:** Correlations between parental acceptance-rejection, power, prestige and child adjustment.

	PARQF	PARQM	POWER	PRESTIGE	PAQ	AGE
PARQM	.49**	—				
POWER	-.27**	.10**	—			
PRESTIGE	-.28**	.14**	.64**	—		
PAQ	.54**	.48**	-.15**	-.08*	—	
AGE	.23**	.20**	-.05	-.01	.28**	—
Mean	37.09	33.25	12.65	14.29	66.19	13.09
SD	10.52	8.83	3.71	3.21	13.55	2.00

*Note*: PARQM = maternal acceptance-rejection; PARQF = paternal acceptance-rejection; PAQ = children’s psychological adjustment.

* *p <* .05

** *p <* .01.

**Table 2:** Hierarchical regression analyses predicting children´s psychological maladjustment adjusted by sex and age.

*Predictors*	*𝛽*	*R*^*2*^	*ΔR*^*2*^
**Step1**		.*08*	.08**
Constant	*40*.*20*		
Sex	.*01*		
Age	29**		
**Step 2**		.*38*	.30**
Constant	*22*.*04*		
PARQF	.37**		
PARQM	.28**		
POWER	*-*.12**		
PRESTIGE	.07*		
**Step 3**		.*39*	.01**
Constant	*3*.*90*		
PARQM*POWER	*-*.*15*		
PARQM*PRESTIGE	*-*.*33*		
PARQF*PRESTIGE	.*23*		
PARQF*POWER	.*04*		

*Note*: PARQM = maternal acceptance-rejection; PARQP = paternal acceptance-rejection

*p < .05

**p < .01

As we can see in Table 2, after adjusting for sex and age (step 1), a first approach to the analysis reveals a significant and positive effect of paternal acceptance-rejection and maternal acceptance-rejection on children’s maladjustment. In addition, there was a significant negative effect of parental power on children’s maladjustment and a significant positive effect of parental prestige on children’s maladjustment; however, the contribution of prestige was the lowest one. No significant interactions were found (step 3). Regarding the contribution of sex and age as co-variables, no significant effect of children’s sex on children’s maladjustment was found, but a significant positive effect of children’s age on children’s maladjustment was found. Because of this, sex was excluded for the subsequent regression analyses conducted for each age group.

### Parental acceptance-rejection, power and prestige predicting children’s psychological adjustment by age group

Considering these previous results, the different effects of the variables were analyzed separately by age group (Table 3). Because gender was not significant, hierarchical regression analyses were only conducted by age groups. Four age groups were analyzed according to different developmental periods: late childhood (9–10 years), early adolescence (11–12 years), mid-adolescence (13–14 years) and adolescence (15–16 years). Independent variables (PARQF and PARQM) were included in the first step to examine direct effects, potential moderators in the second step (Power and Prestige) to test the partial effects of independent variables versus moderators, and finally, in the third step, the product terms of parental acceptance and parental power and prestige were included to examine their moderating and conditional effects.

**Table 3:** Hierarchical regression analyses predicting children’s psychological adjustment by age groups.

Predictors	*𝛽*				*R*^*2*^				*ΔR*^*2*^			
	*G1*	*G2*	*G3*	*G4*	*G1*	*G2*	*G3*	*G4*	*G1*	*G2*	*G3*	*G4*
**Step1**					.*41*	.*57*	.*30*	.*35*	.41**	.57**	.30**	.30**
Constant	*21*.99**	*27*.56**	*34*.25**	*41*.81**								
PARQF	.27**	.35**	.34**	.45**								
PARQM	.46**	.33**	.25**	.21**								
**Step 2**					.*47*	.*57*	.*31*	.*36*	.06**	.*00*	.*01*	.*01*
Constant	*36*.*85*	*30*.24**	*30*.51**	.47**								
PARQF	.*18*	.36**	.41**	.40**								
PARQM	.46**	.34**	.23**	.26**								
POWER	*-*.26**	*-*.*12*	*-*.*11*	*-*.*10*								
PRESTIGE	.*03*	.*05*	.15*	*-*.*01*								
**Step 3**					.*56*	.*60*	.*33*	.*37*	.09**	.03*	.02*	.*01*
Constant	*24*.*92*	*76*.05**	*7*.62**	*36*.23**								
PARQF	*-*.*46*	*-*.*04*	.40**	.50**								
PARQM	*1*.31**	*-*.*03*	.64**	.40*								
POWER	*-*.*08*	.*06*	*-*.*47*	.*06*								
PRESTIGE	.*06*	*-*.78*	.80**	.*05*								
PARQM*POW	.*46*	*-*.*85*	*-*.*14*	.*68*								
PARQM*PREST	*-1*.73**	*1*.26*	*-*.*50*	*-*.*80*								
PARQF*PREST	*1*.42**	.*01*	*-*.*44*	.*52*								
PARQF*POW	*-*.*58*	.*51*	.*53*	*-*.*69*								

*Note*. PARQM = maternal acceptance-rejection; PARQF = paternal acceptance-rejection; POW = parental power; PREST = parental prestige; G1: group from 9 to 10 years old; G2: group from 11 to 12 years old; G3: group from 13 to 14 years old: G4: group from 15 to 16 years old.

*p < .05

**p < .01

Parental acceptance-rejection (by mothers and fathers) showed a significant and negative direct effect on children’s psychological adjustment in all age groups (step 1). However, when the moderators were included, the direct effect of paternal acceptance-rejection was no longer significant for the 9–10-year-old group, and the maternal acceptance-rejection made a greater contribution than paternal acceptance-rejection to the youngest children’s psychological maladjustment (G1). For the rest of age groups the coefficients of maternal acceptance-rejection versus paternal acceptance-rejection were statistically equivalent (confidence intervals at 95%), so maternal rejection and paternal rejection made similar contributions to maladjustment from early adolescence through adolescence.

Regarding the direct effects of interpersonal parental power and prestige, two significant effects were found: a positive effect of parental power on psychological maladjustment at 9–10 years and a negative effect of parental prestige on psychological maladjustment at 13–14 years. Therefore, the results show that the higher the paternal power versus maternal power, the lower the level of maladjustment in late childhood, and the higher the paternal prestige versus maternal prestige, the higher the level of maladjustment in mid-adolescence.

Finally, three two-way significant interactions were found (step 3). Two interactions at ages 9–10: maternal rejection by parental prestige (G1: *𝛽 = -1*.*73*, *t = -2*.*85*, *p = 0*.*00*) and paternal rejection by parental prestige (G1: *𝛽 = 1*.*42*, *t = 3*.*27*, *p = 0*.*00*); and one interaction at ages 11–12: maternal rejection by maternal prestige (G2: *𝛽 = 1*.*26*, *t = 2*.*36*, *p = 0*.*02*). These results show that the relationship between maternal acceptance-rejection and children’s maladjustment was moderated by perceived interpersonal prestige at ages 9–10 and 11–12 years, and relations between paternal acceptance-rejection and children’s psychological maladjustment at ages 9–10.

**Fig 1:**
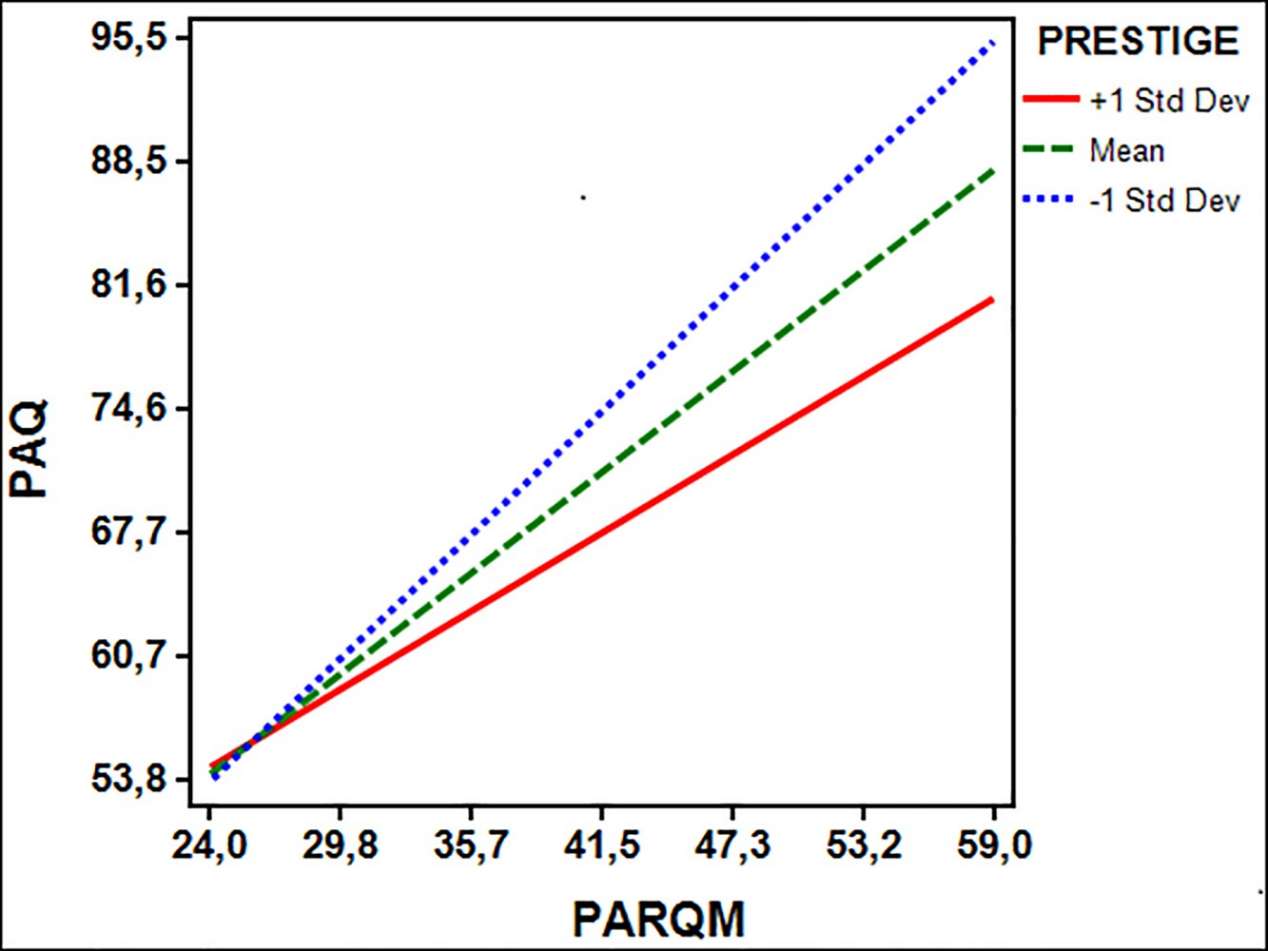
Maternal acceptance-rejection predicting psychological maladjustment at varying levels of prestige in the late childhood (9–10 years old). PAQ = psychological maladjustment (higher scores are indicating maladjustment); PARQM (higher scores are indicating maternal rejection).

**Fig 2:**
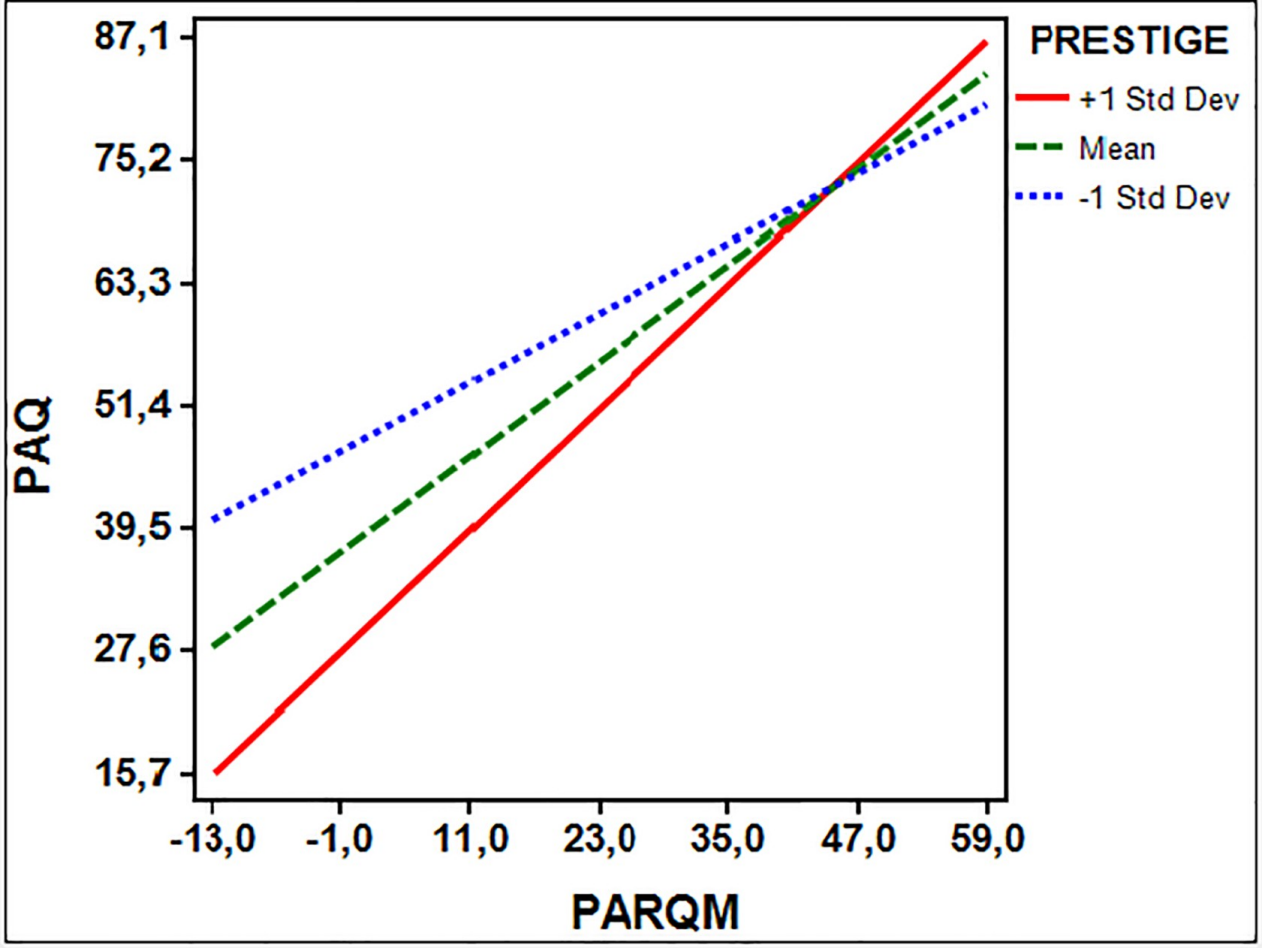
Maternal acceptance-rejection predicting psychological maladjustment at varying levels of prestige in early adolescence (11–12 years old). PAQ = psychological maladjustment (higher scores are indicating maladjustment); PARQM (higher scores are indicating maternal rejection).

The post hoc analysis of the interaction effect of parental prestige on the relations between maternal acceptance-rejection and children’s maladjustment showed different effects in the 9–10-year-old group (Fig 1) versus the 11–12-year-old group (Fig 2). Fig 1 shows that under the condition of +1 SD (mean plus one standard deviation) interpersonal power—where fathers were perceived to have higher prestige than mothers—the effect (simple slope) of maternal acceptance on children’s psychological adjustment tends to be weaker than under the condition of mean (where fathers were perceived to have less prestige than mothers) and under the -1SD condition (where fathers were perceived to have equal prestige with mothers). The higher prestige of mothers over fathers (-1SD condition) intensified the effect of maternal rejection on children’s maladjustment. Higher levels of children’s maladjustment were found when mothers showed both high prestige and high rejection.

In contrast, Fig 2 shows that under the condition of +1 SD (where fathers were perceived to have higher prestige than mothers) the effect of maternal acceptance on children’s psychological adjustment at 11–12 years old tends to be stronger than under the other conditions. Higher levels of maladjustment in early adolescence were found when mothers were viewed with lower prestige than fathers and were also perceived to demonstrate high rejection. According to the Johnson-Newman technique, the moderating effects of prestige on the relations between maternal acceptance-rejection and children’s psychological maladjustment in the 9–10-year-old group is significant for scores below 19.37 on the prestige scale; that is, 19.37 is a transition point at which the moderator variable of prestige is no longer significant within the observed range. Similarly, the transition point in the 11–12-year-old group is 8.7.

**Fig 3:**
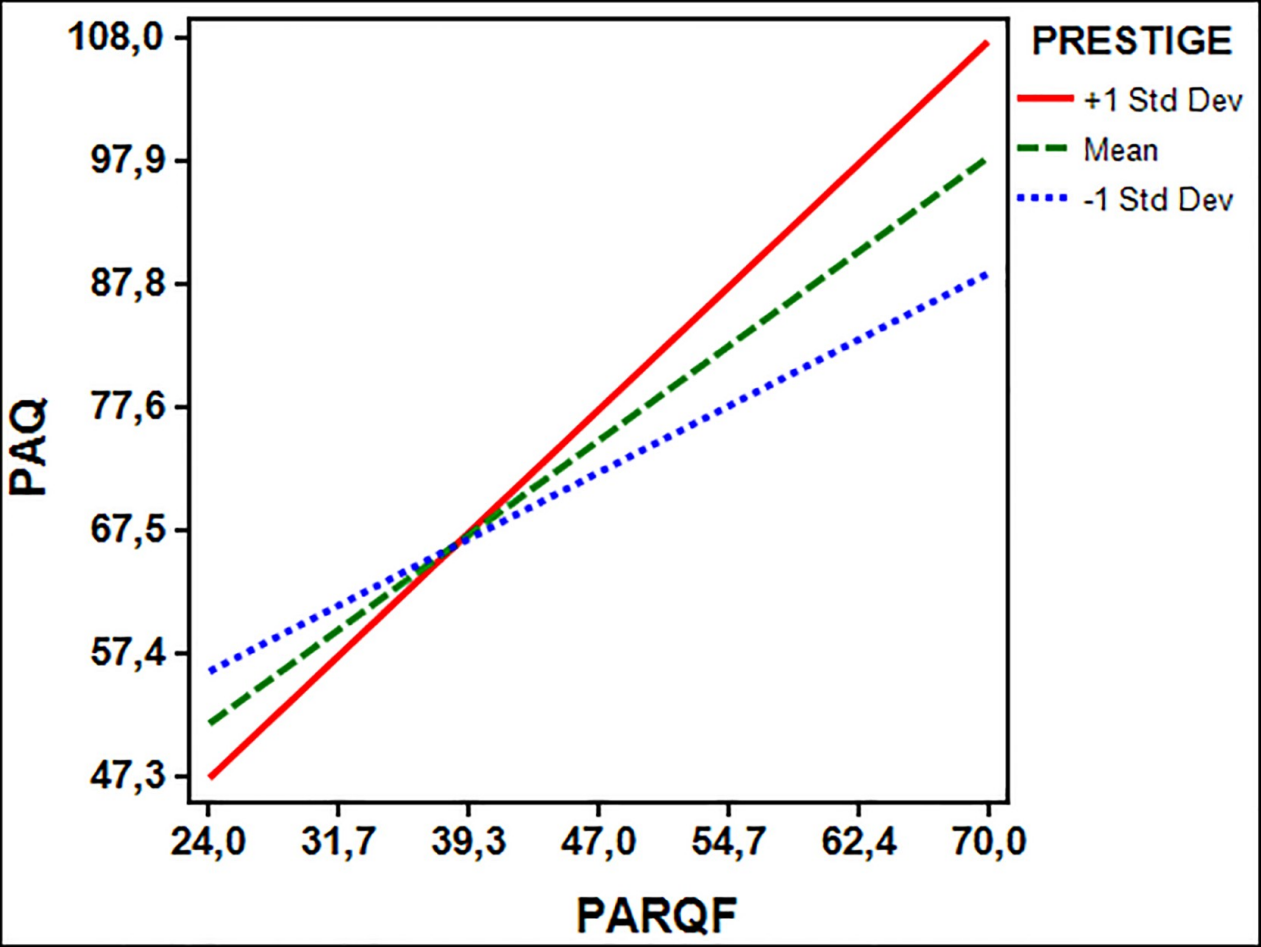
Paternal acceptance-rejection predicting psychological maladjustment at varying levels of prestige in late childhood (9–10 years old). PAQ = psychological maladjustment (higher scores indicate maladjustment); PARQF (higher scores indicate paternal rejection).

Regarding the interaction between paternal acceptance-rejection and parental prestige (Fig 3), the effects (simple slopes in Fig 3) of paternal acceptance-rejection on children’s maladjustment at different levels of prestige in the 9–10-year-old group showed similar results to the moderating effects of prestige on maternal acceptance-rejection at this age. Thus, under the condition of +1 SD interpersonal power—where fathers were perceived to have higher prestige than mothers—the effect (simple slope) of paternal acceptance on children’s psychological adjustment tends to be stronger than under the condition of mean (where fathers were perceived to have less prestige than mothers) and -1SD condition (where fathers were perceived to have equal prestige with mothers). The higher prestige of fathers over mothers (+1SD condition) intensified the effect of paternal rejection on children’s maladjustment. Higher levels of children’s maladjustment were found when fathers showed both high prestige and high rejection. The Johnson-Nyman technique revealed that the moderating effect of prestige is significant for scores above 8.44 on the prestige scale.

## Discussion

The main objective of this study was to learn more about why the acceptance or rejection of one parent could affect a child’s adjustment more than the acceptance or rejection of the other parent when both are significant in the child’s life. From a developmental approach, considering the children’s age and sex, we explore to what extent interpersonal power and prestige might explain the greater impact of one parent on offspring of the other parent from late childhood to adolescence. The results partially support this idea and show how the moderating effect of prestige varies across different age groups.

Preliminary results in this study showed that the children’s sex, in contrast to age, was not significant in predicting psychological maladjustment. For this reason, the variable of sex was excluded from the statistical analyses that followed. Accordingly, recent cross-cultural meta-analyses within the IPARTheory found no differences in the relationship between children’s psychological maladjustment and perceived parental rejection based on a child’s sex [5, 8, 39]. However, these results have not always been consistent (see [26]). Children’s age, on the other hand, was positively related to children’s maladjustment. Thus, our data bring some support to the traditional view of adolescence as a challenging period of life, especially early adolescence [40].

Consistent with previous studies, perceived acceptance-rejection from both fathers and mothers by their children made an independent contribution to the explanation of children's psychological adjustment across different age groups. A great number of studies have shown that perceived parental acceptance- rejection has a significant effect on the psychological adjustment of children and adolescents across different countries and cultures [5–9, 41, 42]. In these studies, it has been also found that perceived acceptance-rejection from both fathers and mothers by their children to explain the children’s adjustment are not always similar in intensity; in some studies perceived paternal acceptance-rejection shows a stronger contribution than maternal acceptance-rejection to explain children’s adjustment [8, 10], while in others the inverse is true [13, 25, 43]. The results in this study showed that perceived maternal rejection, unlike perceived paternal rejection, made a different contribution to explain children’s maladjustment depending on the child’s age. The effect of perceived maternal rejection was higher than the perceived paternal effect at late childhood (age 9–10); however, this difference tended to disappear from early adolescence to adolescence (age 11–16), even as perceived paternal rejection became more relevant, which is consistent with previous studies of these age groups [25, 42, 43]. The higher contribution of perceived maternal rejection in younger children can be explained by the existence of close mother-child relationships at this age. When children become adolescents, they become more distant and demand more autonomy from their parents [27, 44]. It is generally acknowledged that mothers are usually highly involved in the day-to-day care of children during earliest stages of development and spend more time with them than fathers do [45–47].

Regarding the direct effect of parental power and prestige on children’s psychological adjustment, the effect size of power ranged from low to moderate in the younger groups and became non-significant in the adolescent groups. Interpersonal prestige was not significant except in the 13–14-year-old group. Most research has not found significant direct effects of the relative power or prestige of fathers versus mothers on children’s psychological maladjustment (see [24]). However, previous studies with children have shown a significant contribution of interpersonal power [48] or interpersonal prestige [25] to the psychological adjustment of children.

Higher interpersonal power of the father relative to the mother was negatively related to younger children’s maladjustment. Younger children tend to feel very close to their parents; obviously, the parents capacity to influence the decisions and behaviors of children is expected to have a positive effect. Late childhood may be a more sensitive moment for displaying the significance and relevance of the father’s role. As children become more autonomous, fathers may become more involved in their everyday concerns and routines [49]. This shift could probably be supported by maturational and socio-cultural modifications.

In contrast, our data showed that fathers being perceived to have more prestige than mothers is positively related to adolescents’ maladjustment only at 13–14 years. We do not know yet why this was the case. Fathers who show higher signs of social approval, esteem, respect and admiration than mothers might negatively affect early adolescents who interpret this as a kind of superiority or invasive attitude when they need to feel more distance from their fathers [50]. They might also interpret the mother’s lower prestige as a lack of support. In addition, there seems to be a consensus that conflict becomes more intense during early adolescence and less strong from middle to late adolescence [27, 51].

Interestingly, our data showed that the moderating effect of parental prestige on the relations between parental rejection (both mothers and fathers) and children’s adjustment was only significant in the group of younger children, for which maternal rejection made a stronger contribution than paternal rejection to the children’s maladjustment. In this group, the higher prestige of one parent relative to another intensified the acceptance-rejection of the parent who was perceived to have more prestige. Higher levels of children’s maladjustment were found when parents (father or mother) showed both high prestige and high rejection. Likewise, higher levels of adjustment were found when parents showed both high prestige and high acceptance. Prestige seems to be a sign of parental salience that strengthens the impact of the acceptance-rejection by the parents on the child.

The moderating effect of prestige in the group of 11–12-year-olds was only significant for perceived maternal rejection (not perceived paternal rejection); however, the way in which interpersonal prestige affected the relations between perceived maternal rejection and children’s maladjustment was different from the younger group. At 11–12 years old, when the interpersonal prestige of fathers tended to be higher than that of mothers, the impact of perceived maternal rejection on the children’s maladjustment intensified. Higher levels of maladjustment were found when mothers were perceived to have lower prestige than fathers (fathers are perceived to have higher prestige) and mothers were perceived to have high rejection. Consistently, higher levels of early adolescents’ adjustment were found when mothers showed higher perceived prestige than fathers and mothers were perceived to have high acceptance.

Therefore, the significant moderating effects found support the idea that offspring’s perceptions of parental prestige constitute one class of variables that helps to explain the higher influence of one parent over another, and this moderating effect depends on the child’s age. One of the answers to the question of why perceived parental power or prestige moderates this relationship in some instances but not in others [24] may be found in age and its attendant development processes. As has been previously found (see for a review the special issue, [24]) prestige has been a significant moderator in some studies with children or preadolescents (7–12 years old) [25, 43, 52] but not with adolescents (15–17 years old) [43, 48, 53]. Some of these previous studies have shown that interpersonal prestige intensified the effects of paternal rejection [25] and others that it buffered the effects of maternal rejection [42, 53], but no studies to our knowledge have compared these effects across age. The way interpersonal prestige moderates differently in children versus early adolescents is difficult to explain. The higher prestige of one parent versus another may compensate for the effects of their rejection on younger children’s maladjustment due to younger children’s closeness to their parents. For this reason, the perceived lower prestige of one parent versus another does not intensify his/her rejection. In early adolescence this paternal compensation may disappear, and lower maternal prestige may intensify the effects of maternal rejection. On the other hand, we can speculate that interpersonal prestige may have more meaning for adolescents (i.e., they measure prestige based on signs of social approval, esteem, respect and admiration) than for children (i.e., they measure prestige based on signs of salience, regardless of their content), so the lack of maternal prestige could strengthen the effects of maternal rejection on early adolescents’ maladjustment and weaken the effects of maternal rejection on children.

This study has some limitations. First, this research is cross-sectional in design, and we cannot make any causal attributions about the influence of perceived interpersonal power or prestige as moderators of the relationship between perceived parental acceptance and offspring adjustment. For this reason, no causal attributions due to children’s age should be considered. Second, all measures were self-reported and statistical associations obtained may be attributable to shared method variance, so the results should be considered from the children’s perspective. Other perspectives, such as those of parents or external informants, must be considered to confirm these results. Third, the parental power and prestige scale does not allow the power or prestige of one parent to be measured independently from the other’s, so this measure only provides the relative levels of power and prestige between father and mother. Furthermore, this study was conducted in Spain, within a Western cultural context. Different results could be expected in other countries with different cultural contexts and varying roles for fathers and mothers.

For future studies, a longitudinal approach is needed to give a more decisive answer regarding the development of the relationship between parental acceptance-rejection and adolescents’ psychological adjustment, taking in to account the mother and father’s power and prestige. These longitudinal studies should be conducted in different cultural contexts using different sources of information, such as parents or external informants, with independent measures of power and prestige for mothers and fathers.

Despite these limitations, this study has shown that the degree of interpersonal prestige that offspring perceived “catalyzed” the effect of parental rejection on the children’s maladjustment in different ways depending on the child’s age. That is, the results of this study showed that interpersonal parental prestige moderated the relationship between perceived parental (maternal and/or paternal) acceptance-rejection and offspring’s adjustment at late childhood and early adolescence. Thus, the degree of interpersonal prestige may be essential to the intensity of parental acceptance-rejection’s effects on their offspring’s adjustment, especially for younger children and early adolescents.
